# Correlation between the Capillary Blood Flow Characteristics and Endothelium Function in Healthy Volunteers and Patients Suffering from Coronary Heart Disease and Atrial Fibrillation: A Pilot Study

**DOI:** 10.3390/life13102043

**Published:** 2023-10-12

**Authors:** Petr Ermolinskiy, Yury Gurfinkel, Egor Sovetnikov, Andrei Lugovtsov, Alexander Priezzhev

**Affiliations:** 1 Department of Physics, Lomonosov Moscow State University, 1-2 Leninskie Gory, Moscow 119991, Russia; ermolinskiy.pb15@physics.msu.ru (P.E.); anlug1@gmail.com (A.L.); 2 Medical Research and Education Center, Lomonosov Moscow State University, 27-10 Lomonosovsky pr-t, Moscow 119991, Russia; yugurf@yandex.ru (Y.G.); sovetnikov.egor@mail.ru (E.S.)

**Keywords:** capillaroscopy, non-invasive diagnostics, coronary heart disease, atrial fibrillation, optical methods, endothelial function, capillary blood flow velocity, red blood cell aggregation

## Abstract

Coronary heart disease (CHD) and atrial fibrillation (AF) pose significant health risks and require accurate diagnostic tools to assess the severity and progression of the diseases. Traditional diagnostic methods have limitations in providing detailed information about blood flow characteristics, particularly in the microcirculation. This study’s objective was to examine and compare the microcirculation in both healthy volunteers and patient groups with CHD and AF. Furthermore, this study aimed to identify a relationship between blood microcirculation parameters and endothelial function. Digital capillaroscopy was employed to assess the microcirculation parameters, for example, such as capillary blood flow velocity, the size of red blood cell aggregates, and the number of aggregates per min and per running mm. The results indicate significant alterations in blood flow characteristics among patients with CHD and AF compared to healthy volunteers. For example, capillary blood flow velocity is statistically significantly decreased in the case of CHD and AF compared to the healthy volunteers (*p* < 0.001). Additionally, the correlation between the measured parameters is different for the studied groups of patients and healthy volunteers. These findings highlight the potential of digital capillaroscopy as a non-invasive tool for evaluating blood flow abnormalities (red blood cell aggregates and decreased capillary blood flow velocity) in cardiovascular diseases, aiding in early diagnosis and disease management.

## 1. Introduction

Significant research attention has been devoted to the blood microcirculation and capillary blood flow in recent decades. These intricate components of the circulatory system play a pivotal role in ensuring the essential nourishment of tissues with vital substances [1,2]. The microcirculatory system permeates all human tissues and organs. More than 99% of all vessels in the human body are arterioles, capillaries, and venules [3].

Capillaries are the smallest vessels in the body; they connect arteries and veins, and are most closely connected to tissues. There are about 10 billion capillaries in the average human body. They are extremely prevalent in the body; they have a total surface area of about 6300 square meters. As a result, every cell in the body is in close proximity to capillaries, with no cell being more than 50 µm away. Capillary walls are composed of a single layer of cells called the endothelium, which forms the inner lining of all blood vessels. This endothelial layer is extremely thin, allowing for the diffusion of essential molecules like oxygen, water, and lipids to pass through and enter the surrounding tissues [4]. Similarly, waste products such as carbon dioxide and urea can diffuse from the tissues back into the blood through these capillary walls and be eliminated from the body. A “capillary bed” is a network of capillaries located throughout the body. These beds can “open” and “close” at any given time as needed. This process is called autoregulation, and the capillary bed usually carries no more than 25% of the volume of blood it can accommodate at any given time. The more metabolically active cells are, the more capillaries are required to supply them with nutrients [5].

Red blood cells (RBC) are the most abundant cell in the blood, and they can interact with each other under low shear, producing RBC aggregates. In the physiological conditions, the processes of RBC aggregation and disaggregation are in a state of relative equilibrium [6]. However, the mechanisms to maintain the balance in RBC aggregation become insufficient with age [7]. This is especially expressed in patients suffering from coronary heart disease and atrial fibrillation [8].

Coronary heart disease (CHD) is a socially significant pathology, as it is one of the main causes of mortality, as well as temporary and permanent disability of the population in developed countries of the world. Thus, in 2017, in Russia, CHD was detected in 7.8 million adults.

Atrial fibrillation (AF) is a leading cause of illness and death. The main concern is the increased risk of blood clot formation in the left atrial appendage, which can then travel to the brain and cause a stroke. AF raises the risk of stroke by five times and is responsible for 15–20% of all stroke cases [9,10]. Strokes that occur in patients with AF tend to be more severe, resulting in higher mortality rates [11]. As AF primarily affects older individuals (prevalence rates: 10–20% in those aged ≥85 years compared to 0.4–1.0% in those aged 55–60 years), the overall prevalence of AF is projected to more than double by 2050 due to the growing elderly population [12].

Until recently, most of the research in clinical and experimental cardiology was devoted mainly to problems associated with impaired myocardial contractility and perfusion due to socially significant diseases. However, more recently, the increased interest of researchers has been directed to rheologic and microcirculatory disorders in these diseases. For example, our studies have shown that RBC aggregation is elevated in CHD patients [8].

During the last few decades, laser Doppler flowmetry has emerged as an efficient technique for studying the blood perfusion and microcirculation disorders in different diseases [13]. Recently, portable devices that enable the researchers to conduct measurements in various experimental conditions, including microgravity, have been designed [14]. Additionally, a variety of interesting studies of phenomena related to blood and lymph microcirculation in the brain of laboratory animals have been performed using the speckle correlation microscopy technique [15,16].

One of the methods of lifetime study of blood microrheological properties is digital capillaroscopy, a method of optical non-invasive microscopy that allows for the direct visualization of superficial skin microvessels and their surrounding tissue in vivo. Image processing software enables the non-invasive quantitative assessment of static and dynamic parameters of microcirculation [17,18]. Capillaroscopy allows for the quantitative assessment of important factors such as capillary network density, the level of perivascular edema, and the size of capillary cross-sections. Furthermore, capillaroscopy enables the measurement of capillary blood flow velocity and the visualization of blood aggregates within the capillaries. Additionally, the diffuse light scattering method can be employed to measure RBC aggregation parameters in vitro alongside with capillaroscopy [19,20]. The diffuse light-scattering technique involves registering and analyzing the intensity of light scattered from a layer of whole blood [21,22,23].

Capillaroscopy is a widely used diagnostic tool for distinguishing between Raynaud’s syndrome and connective tissue diseases, specifically systemic sclerosis [24]. It is also utilized for assessing the severity of capillaroscopic changes and their association with autoantibodies to scleroderma antigens, particularly Scl-70 [25]. Recently, a comprehensive review discussed the correlation between microvascular abnormalities, endothelial dysfunction, and internal organ changes in systemic scleroderma [26]. This review highlighted the importance of microcirculation studies in conjunction with other morphological and functional approaches to better understand and monitor microcirculatory deterioration in systemic sclerosis, as well as assess disease progression and the response to treatment.

The capillary network with its complex system of regulation is composed of endothelium, which plays, as established by numerous studies, a role that is far from passive [4,27,28]. The independent role of the endothelium in the regulation of vascular tone was first reported in a paper by Furchgott and Zawadzki published in *Nature* in 1980 [29]. The authors discovered the ability of an isolated artery to change its muscle tone in response to acetylcholine without the involvement of central (neurohumoral) mechanisms. The main merit in this was attributed to endothelial cells, which were characterized by the authors as the cardiovascular «endocrine organ» that communicates between blood and tissues [30].

In 1998, F. Murad, R. Furchgott and L. Ignarro received the Nobel Prize in Medicine for their discovery of the role of nitric oxide as a signaling molecule in the regulation of the cardiovascular system. The authors found that nitroglycerin and similar drugs act as vasodilators as a result of their conversion, with endothelial involvement, to nitric oxide (NO), which has a major relaxing effect on blood vessels. In addition, NO molecules inhibit key links in the development of atherosclerosis, such as platelet adhesion and aggregation, leukocyte adhesion and migration, and smooth muscle cell proliferation [31].

Nitric oxide was found responsible for neuron signal transduction, immune reactions, reproductive functions, etc. Not only NO but also its numerous derivatives can manifest this activity. Among them peroxynitrite, nitrites, nitrates, nitrosothiols, and heme- and non-heme nitrosyl complexes occupy a specific place. Except for peroxynitrite, all of them can release free NO and thus play a role of NO de-pot. The generation and decay mechanisms of these substances, in particular nitrosyl complexes, and their biological activity have been clarified [32]. Nitric oxide functions as a signaling molecule in almost all organs and tissues of animals and humans. In the 1980s, effective NO traps were proposed, capable of accumulating it in the body for 1–2 h, followed by reliable registration. Such traps turned out to be complexes of ferrous iron with derivatives of dithiocar-bamate. By bonding with them, NO forms paramagnetic mononitrosyl iron complexes, which are detected using the electron paramagnetic resonance method. Along with the incorporation of NO into exogenous iron-containing traps, it can form paramagnetic dinitrosyl iron complexes in cells and tissues, which include endogenous iron and thiol (sulfur-containing) groups of proteins and low-molecular-weight compounds [33]. Recently, an important role of other gastransmitters (CO and H_2_S) has been identified in the regulation of blood circulation and microrheology [34].

The theoretical basis for a new direction of fundamental and clinical research was created, i.e., the study of the role of endothelial dysfunction in the pathogenesis of cardiovascular diseases and the search for ways to effectively correct it. Prostacyclin and endothelial hyperpolarizing factors (EDHF), i.e., a generic term for substances and signals that hyperpolarize vascular myocytes by opening voltage-dependent channels, also play an important role in the microcirculation [27,35].

The endothelium has emerged as the key regulator of vascular homeostasis, in that it has not merely a barrier function but also acts as an active signal transducer for circulating influences that modify the vessel wall phenotype [36]. In clinical practice, endothelial dysfunction is primarily associated with the deterioration of the capillary blood flow, which is unable to adequately provide the necessary metabolism between blood and tissues. It is important to note that most cardiovascular risk factors are associated with endothelial dysfunction [37]. Alteration in endothelial function (EF) precedes the development of morphological atherosclerotic changes and can also contribute to lesion development and later clinical complications [38]. Atherosclerosis begins in childhood, quietly progresses through a long preclinical stage, and finally manifests clinically, usually in middle age. Over the past 30 years, it has become clear that the onset and progression of the disease, as well as its subsequent activation, which increases the risk of pathologic events, depend on profound dynamic changes in vascular biology [39].

Evidence to date suggests that EF serves as an integrative indicator of the combined impact of both traditional and novel risk factors on the arterial wall, as well as its capacity for repair. This endothelium-dependent vascular biology plays a crucial role not only in the development and advancement of atherosclerosis but also in the shift from stable to unstable disease states accompanied by heightened risks. Consequently, investigating EF in clinical trials has emerged as a significant endpoint, supplementing the assessment of circulating risk factors, imaging techniques for structural artery disorders (e.g., carotid intima-media thickness, intravascular ultrasound, and computed tomography), and traditional cardiovascular clinical outcomes [38].

Nowadays, there are several methods allowing researchers to quantify the endothelium function parameters. For example, the authors in [40] reported a non-invasive ultrasound test to assess the vascular function of the conduit arteries in the systemic circulation. In this approach, the diameter of the brachial artery is measured before and after an increase in shear stress, inducing hyperemia. When a sphygmomanometer cuff placed on the forearm distal to the brachial artery is inflated to 200 mmHg and then released after 4–5 min, hyperemia occurs primarily as a result of local endothelial NO release [41]. However, there are practical difficulties that must be overcome before this technique is suitable for use in routine clinical practice [42]. These challenges include the need for highly skilled operators, expensive equipment, and the care required to minimize the effects of environmental or physiological factors such as exercise, food intake, caffeine, and significant temperature fluctuations. Hyperemia is also determined in part by the magnitude of postischemic vasodilation, making it a measure of microcirculatory function [43].

The aim of this study was to investigate and compare the microcirculation parameters in healthy volunteers and groups of patients with CHD and AF. Additionally, the purpose of the study was to find the correlation between the parameters of blood microcirculation and EF, which, to the authors’ knowledge, has not been previously performed.

## 2. Materials and Methods

### 2.1. Patients

This study of microcirculation involved 132 adults, including 44 healthy volunteers, 44 patients with CHD and 44 patients with AF (Table 1). Overall, three groups are presented for comparison: control group (healthy volunteers), group of patients with CHD, and group of patients with AF.

An additional study of the correlation between microcirculatory parameters and EF included 47 adults extracted from an initial sample of 132 adults for whom EF was measured (Table 2).

Patients with AF, as well as CHD patients presented in this study, take oral anticoagulants (mainly apixaban and rivaroxaban) according to the protocol adopted by the cardiology department.

The exclusion criteria were the following: patients over 85 years of age, patients with oncological pathology, chronic liver and kidney disease, the presence of type 2 diabetes, pathology of the valvular apparatus of the heart, connective tissue diseases or diseases of the central nervous system. In addition, patients with both CHD and AF were excluded from this study.

The study design was approved by the Ethics Committee of Lomonosov Moscow State University Medical Research and Education Center (#11/22 from 5 December 2022). The patients and healthy volunteers participating in this study were informed of the purpose of the research and gave written informed consent in accordance with the Declaration of Helsinki.

### 2.2. Vital Digital Capillaroscopy

**Figure 1:**
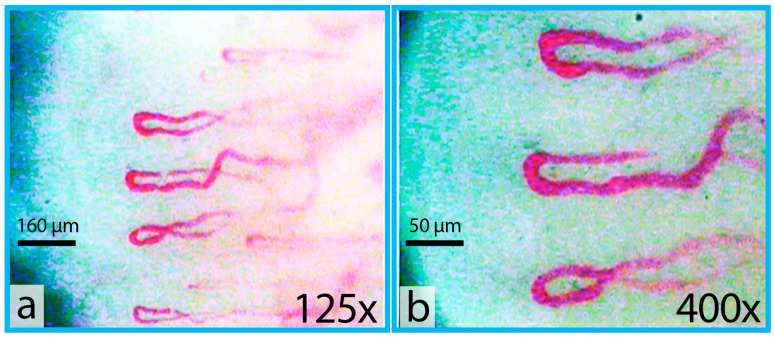
Example images of capillaries of the nail bed of a healthy 74-year-old man at (**a**) 125× magnification and (**b**) 400× magnification.

The digital capillaroscope Kapillaroskan-1 (AET, Moscow, Russia) was utilized to visualize the nail fold capillaries. It was equipped with a high-speed CCD-camera (1/3″ monochrome progressive scan IT CCD sensor, resolution 640 × 480 px, frame rate 200 fps full frame), specifically the TM-6740GE (JAI, Akishima, Japan). The illumination of the nail bed was achieved using an LED-based illuminating system. Two sets of total magnification were employed: 125× and 400×. The 125× magnification was used to capture panoramic images of the capillaries (Figure 1a), facilitating the selection of higher quality images among capillaries in the nail bed, while the 400× total magnification allowed for more detailed imaging of individual capillaries (Figure 1b). This higher magnification also facilitated the measurement of static parameters such as capillary length and diameter in various parts of the capillary, as well as the assessment of capillary blood velocity in different capillary segments.

The procedure of the measurements was as follows. After a minimum of 15 min of sitting and resting of an individual, the measurements of his/her microcirculation parameters were carried out in a temperature-controlled room between 9 and 11 am. The room temperature was maintained between 22 and 23.5 °C. The participant was seated with his/her left hand at heart level. To ensure accurate results, all participants were instructed not to smoke or consume caffeine-containing beverages for at least one day prior to the examination. The skin in eponychium of the finger prepared for the study was treated with 70% alcohol solution in order to remove fat and contaminants from it. After about 3–5 min, 1–2 drops of cedar oil were applied to the skin to optically clear the eponychium. Skin temperature was measured at the dorsal middle phalangeal area of the tested finger using the AND DT-635 skin thermometer (A&D, Akishima, Japan). The average skin temperature was 33.4 ± 1.5 °C and there were no statistically significant differences observed among the individuals in the studied groups. The video of blood flow in capillaries was taken in the eponychium of either the fourth or third finger of the left hand. In the case of insufficient quality visualization, this study was also performed on the eponychium of the second and/or fifth finger.

**Figure 2:**
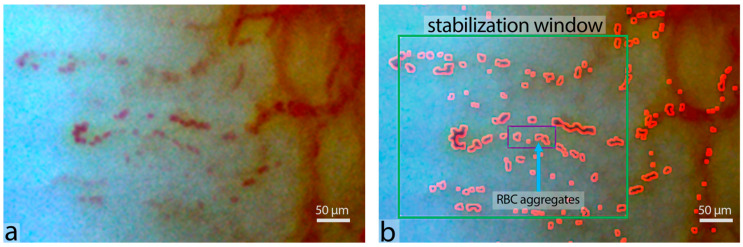
(**a**) Example image of capillaries of the nail bed of an 82-year-old patient not on anticoagulants. (**b**) Program processing of the image is presented: selection of aggregates and determination of their number in the counting window.

In this study, for the first time, we used our advanced automatic program for image processing of nail bed capillaries based on complex image analysis. The algorithm of analysis consists of three steps: (1) stabilization of the video stream and noise suppression in the image; (2) cumulative construction of the capillary shape and determination of its compartments; and (3) straightening out of the capillary image into a linear form, enabling the calculation of both the number of aggregates and their velocities. The examples of raw image and processed image with the selection of RBC aggregates are shown in Figure 2. Only the videos with 400× magnification were processed using the automatic program. The duration of one video was about 5 sec and 4–5 videos were recorded for each individual to obtain 10–12 capillaries in total. In the presence of stasis or pathology, the number of recorded videos, and, consequently, the capillaries was increased.

In our previous studies, we only considered visually the presence or absence of aggregates in the studied capillaries [20]. The new program allows us to quantitatively estimate the size of RBC aggregates (their area in μm^2^), the number of aggregates per min and the number of aggregates per running mm (Figure 2), as well as capillary blood flow velocity in each capillary. This makes it possible to significantly increase the accuracy of the study, to carry out rapid processing of the data, and allows the method to be used for comparative studies. Overall, four parameters of microcirculation were measured:

- Size of RBC aggregates—the average area of RBC aggregate in μm^2^.
- Number of aggregates per min—ratio of the number of observed aggregates in a capillary per measuring interval. The aggregate count is calculated by the number of crossings of a fixed capillary cross section.
- Number of aggregates per running mm—the average number of observed aggregates in a capillary over the linear dimension of the capillary during measuring interval.
- Capillary blood flow velocity—the averaged velocity of moving single RBCs and RBC aggregates determined in the observed capillary during measuring interval in μm/s.

Finally, these parameters were calculated as the mean value measured on at least 10 capillaries from each patient with CHD or AF and on at least 6 capillaries from a healthy donor in the Control group.

The example videos of capillary blood flux are presented in the Supplementary Materials section for a patient with CHD (Video S1) and for a healthy individual (Video S2).

### 2.3. Characterization of the Endothelium Function

In addition, we studied EF in groups of healthy volunteers and patients with CHD and AF (Table 2). For this purpose, we used an experimental device “Angiochek” (Russia), the design feature of which is the use of highly sensitive acoustic sensors located in the projection of the brachial artery and wrist arteries.

EF was determined using a method similar to that described in the literature as the method of pulse tonometry [44]: within 30 s using a cuff placed on the wrist and connected to a sensor, the amplitudes of pulse waves of the wrist arteries were automatically measured. Then, air was pumped into the cuff placed on the upper arm at a pressure exceeding the patient’s systolic blood pressure by 30–40 mmHg. At the end of the three-minute hyperemia, the amplitudes of pulse wave amplitudes on the wrist arteries were also automatically measured again within 60 s. At the same time EF was defined as the ratio of pulse wave amplitudes after and before constriction, expressed as a percentage. Normally, the pulse wave amplitude growth after the occlusion test is approximately 30% according to this technique. In this study, only the EF of wrist was considered.

### 2.4. Statistical Analysis of the Data

All data were processed, and all figures were plotted using applications developed on Python. The figures with box plot representation of the measured parameters consist of the box extending from the first quartile (Q1) to the third quartile (Q3) of the data, with a median line. The whiskers on figures correspond to the standard deviations with the mean values in the centers.

The correlations between different parameters were estimated using Pearson’s correlation coefficient. The correlation is considered weak if the Pearson’s coefficient is 0.3≤|r|<0.5 and strong if the Pearson’s coefficient is |r|≥0.5.

The statistical significance of the differences between samples was calculated using the Mann–Whitney U test. Two samplings were considered statistically significantly different if the *p*-value was lower than 0.05 (* *p* < 0.05; ** *p* < 0.01; *** *p* < 0.001).

## 3. Results

### 3.1. Microcirculation in Studied Groups

#### 3.1.1. Microcirculation Parameters

**Figure 3:**
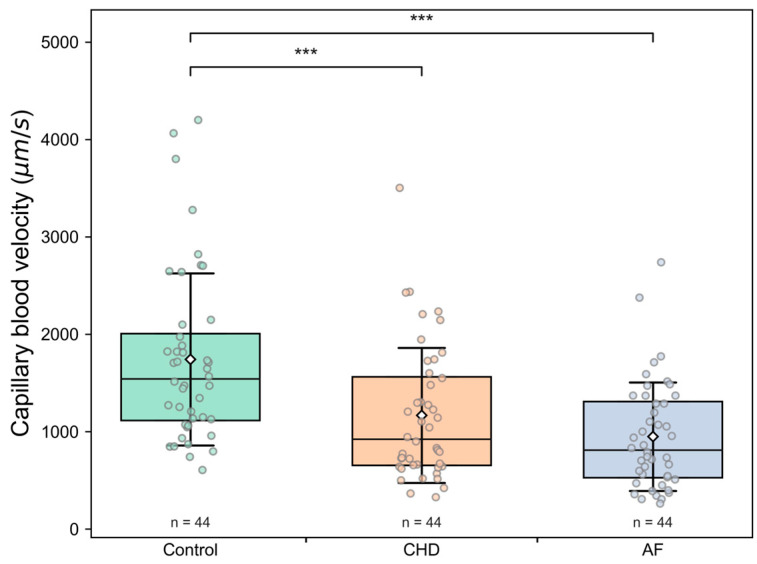
The average capillary blood flow velocity for three groups: Control (healthy individuals), CHD (coronary heart disease) patients and AF (atrial fibrillation) patients. Each point in the figure corresponds to the average value for a single donor or patient. The box corresponds to the first (Q1) and to the third quartile (Q3) of the data with a median line. The whiskers are standard deviation. The rhombus point is the mean value. *** *p* < 0.001.

**Figure 4:**
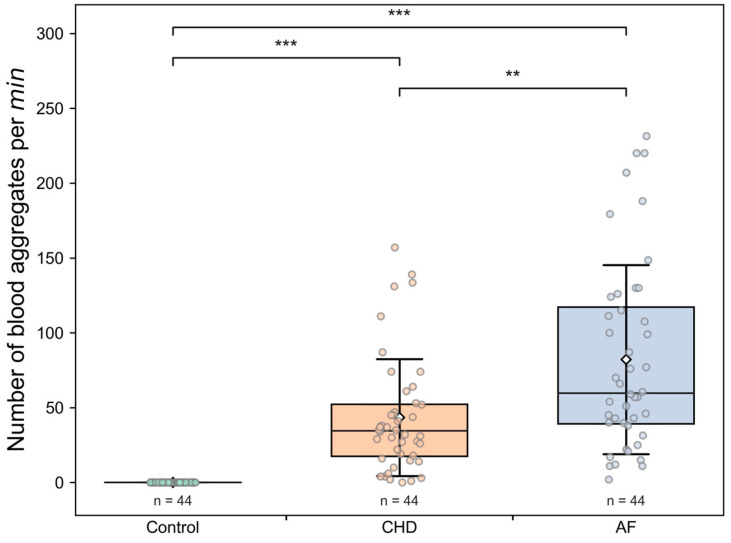
The number of blood aggregates per min for three groups: control (healthy individuals), CHD (coronary heart disease) patients and AF (atrial fibrillation) patients. Each point in the figure corresponds to the average value for a single donor or patient. The box corresponds to the first (Q1) and to the third quartile (Q3) of the data with a median line. The whiskers are standard deviation. The rhombus point is the mean value. ** *p* < 0.01; *** *p* < 0.001.

**Figure 5:**
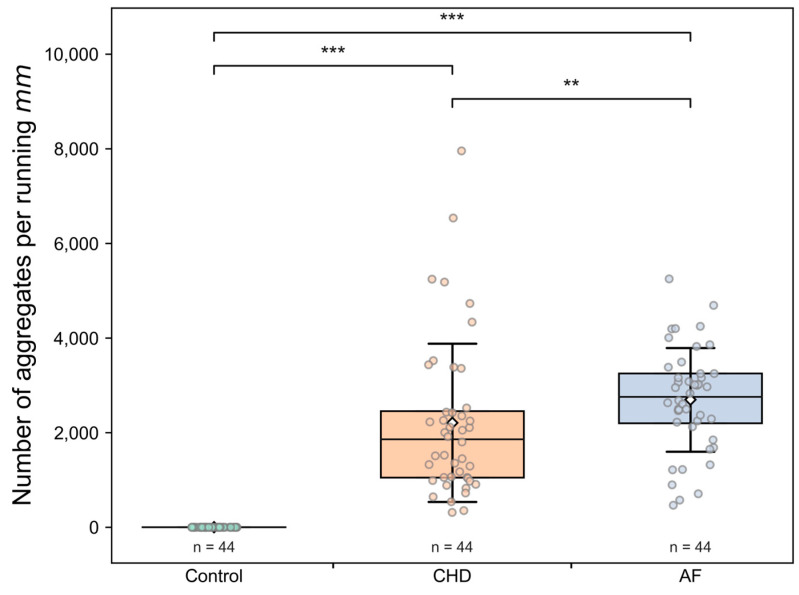
The number of blood aggregates per running mm for three groups: control (healthy individuals), CHD (coronary heart disease) patients and AF (atrial fibrillation) patients. Each point in the figure corresponds to the average value for a single donor or patient. The box corresponds to the first (Q1) and to the third quartile (Q3) of the data with a median line. The whiskers are standard deviation. The rhombus point is the mean value. ** *p* < 0.01; *** *p* < 0.001.

The comparison of the microcirculation parameters between Control, CHD and AF group is presented in Table 3 and in Figure 3, Figure 4 and Figure 5. The average capillary blood flow velocity is statistically significantly higher (*p* < 0.001) in the control group compared to that the CHD and AF groups (Figure 3). There are no statistically significant differences in capillary blood flow velocity between CHD and AF group (Figure 3). However, statistically significant differences between the CHD and AF group are observed (*p* < 0.01) for the number of blood aggregates per min (Figure 4) and for the number of blood aggregates per running mm (Figure 5). There are no observed RBC aggregates in the control group (Figure 4 and Figure 5). Interestingly, the pathological alterations are expressed very well in the parameters characterizing RBC aggregates. The parameter of blood aggregate size is not statistically different between the CHD and AF group (see Figure S1).

To summarize, these facts explicitly show that an increase in all measured characteristics of RBC aggregation in the studied diseases leads to an essential decrease in the capillary blood flow velocity.

In the control group, there is a wide range of different ages compared to the CHD or AF group (Table 1). It was shown that there is no correlation between the average blood flow velocity and the individuals’ ages (Figure S2).

#### 3.1.2. Correlations between the Parameters of Microcirculation

**Figure 6:**
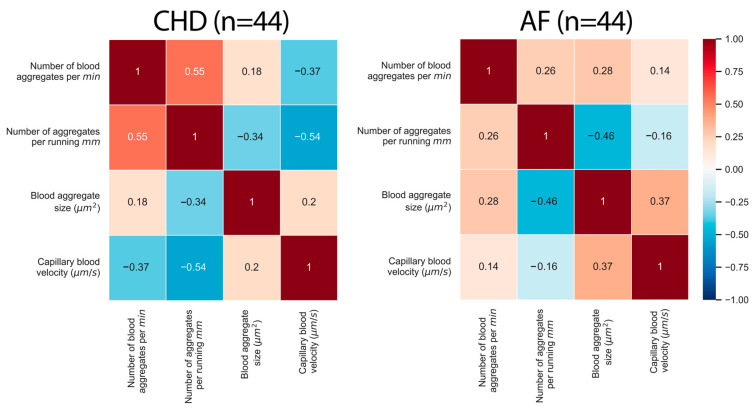
The correlation matrix of microcirculation parameters for two groups of patients: (**left**) CHD (coronary heart disease) and (**right**) AF (atrial fibrillation). The correlations between parameters are characterized by the Pearson’s correlation coefficient.

**Figure 7:**
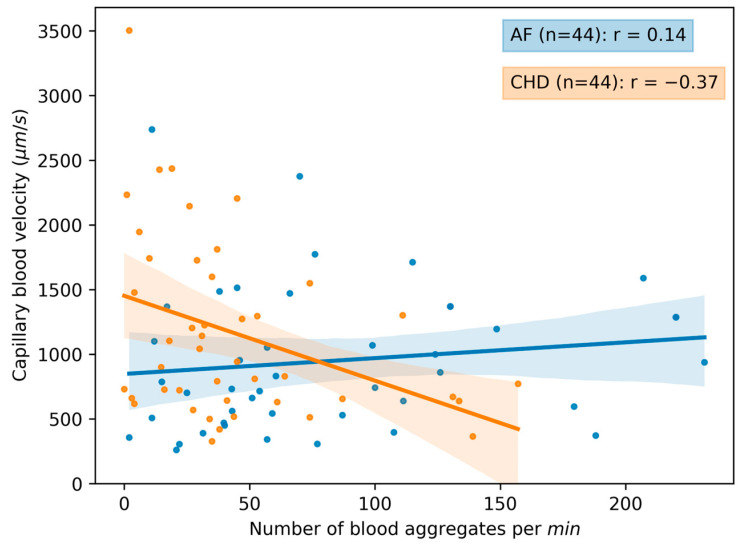
The scatterplot and correlation between the capillary blood velocity and the number of blood aggregates per min for 2 groups of patients: CHD (coronary heart disease) and AF (atrial fibrillation). Data for volunteers in the control group are omitted due to the absence of observed aggregates in the bloodstream. Each point in the figure corresponds to the average value for a single donor or patient. The linear regression of the data and its errors for each group is presented. Pearson’s correlation coefficient is calculated for each group and given in the legend.

**Figure 8:**
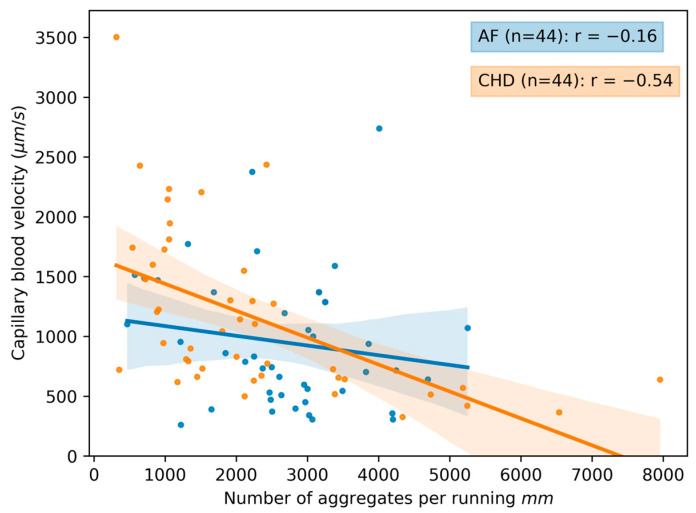
The scatterplot and correlation between the capillary blood velocity and the number of blood aggregates per running mm for two groups of patients: CHD (coronary heart disease) and AF (atrial fibrillation). Data for volunteers in the control group are omitted due to the absence of observed aggregates in the bloodstream. Each point in the figure corresponds to the average value for a single donor or patient. The linear regression of the data and its errors for each group is presented. Pearson’s correlation coefficient is calculated for each group and given in the legend.

**Figure 9:**
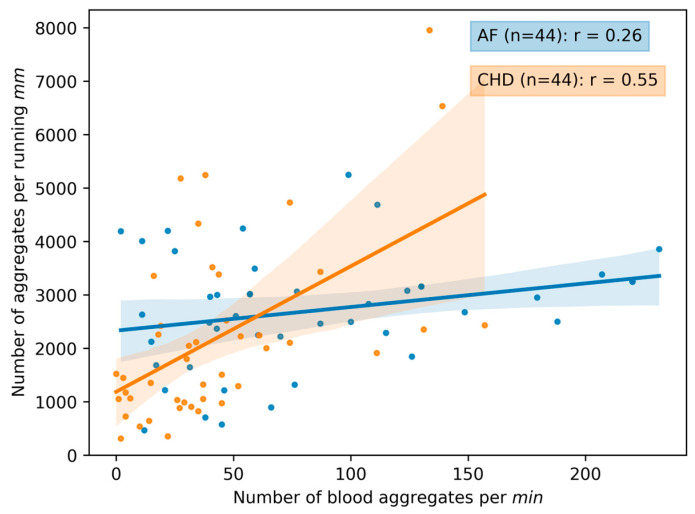
The scatterplot and correlation between the number of blood aggregates per running mm and the number of blood aggregates per min for two groups of patients: CHD (coronary heart disease) and AF (atrial fibrillation). Data for volunteers in the control group are omitted due to the absence of observed aggregates in the bloodstream. Each point in the figure corresponds to the average value for a single donor or patient. The linear regression of the data and its errors for each group is presented. Pearson’s correlation coefficient is calculated for each group and given in the legend.

In Figure 6, the correlation matrixes are presented for the AF and CHD groups. The correlation curves between the microcirculation parameters are presented in Figure 7, Figure 8 and Figure 9. One can see that there are certain differences between the correlation coefficients of microcirculation parameters for these two groups, e.g., the capillary blood flow velocity inversely correlates with the number of aggregates per running mm for the CHD group (r=−0.37), whilst there is no correlation for these parameters for the AF group (r=0.14) (Figure 6).

This fact may be due to the difference in the nature of the studied diseases: while CHD disorders primarily originate from the disorders of the microcirculatory system, AF disorders originate from the disorders in the heart muscle contractions.

A different kind of correlation patterns for different parameters is presented in Figure 7, Figure 8 and Figure 9. It is evident that there is a stronger correlation among the microcirculation parameters in the CHD group when compared to the AF group. Interestingly, there is an inversed correlation between the capillary blood velocity and the number of blood aggregates per min (r=−0.37) in the group of CHD patients (Figure 7) as well as between the capillary blood velocity and the number of blood aggregates per running mm (r=−0.54) in the group of CHD patients (Figure 8). This may indicate that the increase in RBC aggregates may act as a compensatory mechanism.

In Figure 9, the number of blood aggregates per running mm correlates with the number of blood aggregates per min predominantly in CHD patients (r=0.55) compared to patients with AF (r=0.26). This may indicate a greater involvement of the cardiovascular system in the regulation of the microcirculation process, since peripheral arteries in CHD patients are also affected like coronary arteries, although to a lesser extent.

Additional correlation curves are presented in Figures S3–S5. The capillary blood velocity correlates with the blood aggregate size in patients with AF (r=0.37) whereas the correlation of these parameters for patients with CHD is not as significant (r=0.20) (Figure S3). It can be assumed that this is due to the compensatory response of the body to improve the flow of aggregates through microvessels, i.e., the larger the size of RBC aggregates, the higher blood flow velocities are required. The blood aggregate size inversely correlates with the number of aggregates per running mm for both groups (r<−0.3) (Figure S4). This is because larger aggregate sizes result in a decrease in the number of aggregates present in the capillary. However, the blood aggregate size does not change significantly with changes in the number of aggregates per min for both groups (Figure S5).

### 3.2. The Correlation between Microcirculation Parameters and the Endothelial Function

Microcirculation parameters and EF measured on the wrist for the patients and healthy volunteers are presented in Table 4. The negative values of Mean−SD for EF measured on the wrist for CHD and AF groups are due to the narrowing of the arteries instead of the opening of the arteries after the artery occlusion.

**Figure 10:**
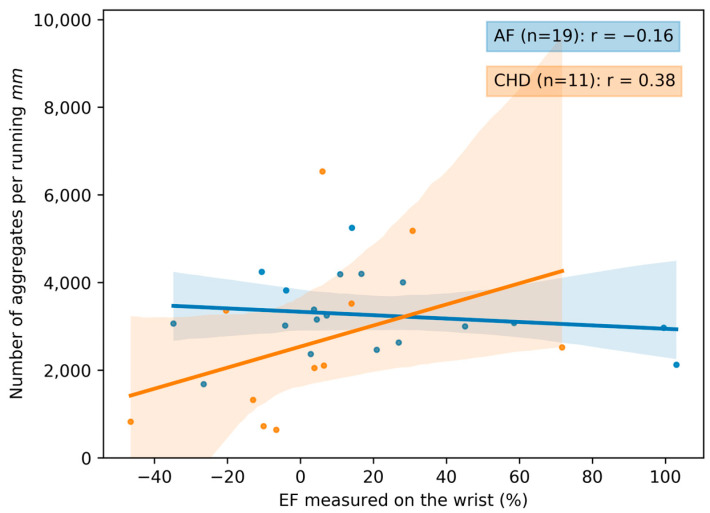
The scatterplot and correlation between the number of blood aggregates per running mm and the EF (endothelial function) measured on the wrist for two groups of patients: CHD (coronary heart disease) and AF (atrial fibrillation). Data for volunteers in the control group are omitted due to the absence of observed aggregates in the bloodstream. Each point in the figure corresponds to the average value for a single donor or patient. The linear regression of the data and its errors for each group is presented. Pearson’s correlation coefficient is calculated and given in the legend.

**Figure 11:**
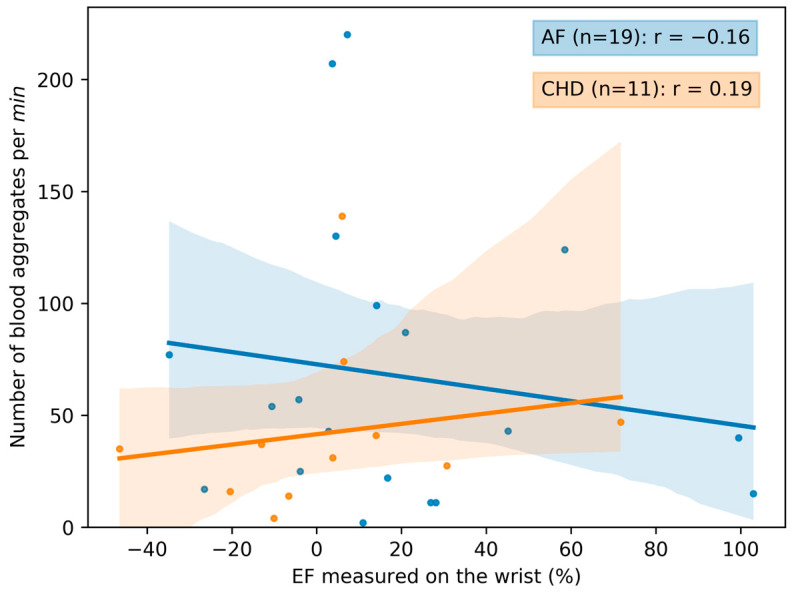
The scatterplot and correlation between the number of blood aggregates per min and the EF (endothelial function) measured on the wrist for two groups of patients: control (healthy individuals), CHD (coronary heart disease) and AF (atrial fibrillation). Data for volunteers in the control group are omitted due to the absence of observed aggregates in the bloodstream. Each point in the figure corresponds to the average value for a single donor or patient. The linear regression of the data and its errors for each group are presented. The corresponding Pearson’s correlation coefficients are calculated and given in the legend.

The correlation curves between the microcirculation parameters and the endothelial function are presented in Figure 10 and Figure 11. Additional correlation curves are presented in Figures S6 and S7. The correlation between the microcirculation parameters and the endothelial function is higher for the patients with CHD. Specifically, an increase in EF measured on the wrist leads to the significant decrease in the blood aggregate size (Figure S7). The weaker correlations for the AF group are most likely due to the fact that the peripheral vasculature is less affected in AF. However, due to the small size of the groups with the EF parameter, it is not possible to make definitive statements.

## 4. Discussion

In recent years, due to the emergence of new technological capabilities and advances in video image processing, it has been possible to significantly expand the scope of application of digital non-invasive capillaroscopy for the needs of scientific and practical medicine. Pathologic changes in the capillaries of the nail bed reflect the pathology of the cardiovascular system [17], tissue diseases [45], as well as the presence of diabetes mellitus [46]. The results obtained in this study clearly demonstrate that the possibilities of nail bed capillaroscopy are not only not exhausted, but in fact, only open up new perspectives for personalized assessment in the administration of medications such as oral anticoagulants.

Two groups of patients were compared—those with CHD and AF, despite different pathophysiologic causes. Our preliminary studies have revealed that both patient groups exhibit observable red blood cell aggregates in capillaries, reduced capillary blood flow velocity, and impaired EF assessed through pulse tonometry at the wrists, as compared to healthy volunteers. Additionally, patients from both groups underwent the same oral anticoagulant treatment. Given these observations, we aimed to investigate potential correlations between these microcirculatory and EF parameters.

For the first time in this study, it was possible to show not only the possibility of determining RBC aggregates circulating in capillaries, but also to determine their number per unit area and quantify them per unit time. In healthy volunteers under room-temperature conditions, as shown in our study, RBC aggregates were absent. Apparently, this is the synergistic result of the coordinated work of endothelial cells, their glycocalyx and glycocalyx of RBCs themselves. At the same time, in CHD and AF, we observe a significant number of aggregates in the capillary bed (Figure 4 and Figure 5). The number of aggregates per running mm is significantly higher in patients with AF than in patients with CHD (Figure 5), and the mean values of capillary blood flow velocity are significantly lower in patients with AF (Figure 4). The results showed an insufficient therapeutic effect of oral anticoagulants on blood microcirculatory parameters in patients with AF or CHD.

It should be noted that an increase in the number of aggregates per running mm in patients of both groups is accompanied by a decrease in the capillary blood flow velocity. This correlation is more pronounced in patients with CHD (Figure 8), which is apparently due to the fact that the disorders of the microcirculatory system are the primary source of CHD disorders. Additionally, EF in CHD is farther from normal values (in comparison with the control group) than in AF. This might be because AF primarily affects the heart, while in CHD, the pathological process involves both the coronary arteries and the peripheral arteries. At the same time, the increase in the number of aggregates per min and per running mm, as well as the reduction in capillary blood flow velocity, are expressed to a greater extent in AF. This suggests that the decrease in EF affects the formation of aggregates to a lesser extent than the chaotic heart rhythm (see Table 4).

However, this study has the following limitations. First, the control and patient groups of this pilot study were rather small due to strict criteria of exclusion of initial candidates for participation in the study (see above). However, even the results obtained on the groups of 44 individuals each allow for the drawing of preliminary conclusions regarding the relationships between the parameters measured for the first time in parallel in vivo characterizing different aspects of microcirculation. Evidently, the inclusion of additional data to be obtained on larger study groups will enable us to decrease the statistical scattering of the data and obtain more precise information about the studied correlations. Second, being limited in the number of individuals in the study groups, we were unable to differentiate between the individuals of different ages; however, it is known that some alterations in the microcirculation and endothelial function parameters are age-associated. An increase in the number of individuals in study groups will enable us to differentiate the alterations of the studied parameters and their correlations due to age and due to the specificity of the diseases.

## 5. Conclusions

The significance of this study is due to the potential benefits it can bring to personalized medicine. By employing the non-invasive in vivo control methods, we can not only determine the presence of RBC aggregates in the capillary bed, but also assess their quantitative parameters. This information will greatly assist practicing physicians in adjusting the treatment with thrombolytic drugs for patients with CHD and AF without requiring excessive amounts of time.

## Figures and Tables

**Table 1:** Sample of groups in which microcirculation measurements were performed.

Parameter	Control	CHD ^1^	AF ^2^
Number of individuals	44	44	44
Number of females	19	14	25
Number of males	25	30	19
Mean age ± SD (years)	36.5 ± 18.5	70.4 ± 8.7	73.3 ± 9.9

^1^ coronary heart disease; ^2^ atrial fibrillation.

**Table 2:** Sample of groups in which microcirculation and endothelium function measurements were performed simultaneously.

Parameter	Control	CHD ^1^	AF ^2^
Number of individuals	17	11	19
Number of females	6	2	9
Number of males	11	9	10
Mean age ± SD (years)	39.3 ± 22.4	69.9 ± 6.9	74.1 ± 9.3

^1^ coronary heart disease; ^2^ atrial fibrillation.

**Table 3:** Microcirculation parameters for three groups (see Table 1): control, CHD and AF. The mean values and standard deviations of the data are presented.

Parameter	Control (N = 44)	CHD ^1^ (N = 44)	AF ^2^ (N = 44)
Capillary blood velocity	1742 ± 884	1168 ± 694	949 ± 557
Blood aggregate size µm^2^	0 ± 0	104 ± 35	111 ± 35
Number of aggregates per running mm	0 ± 0	2208 ± 1673	2696 ± 1096
Number of blood aggregates per min	0 ± 0	43 ± 39	82 ± 63

^1^ coronary heart disease; ^2^ atrial fibrillation.

**Table 4:** Microcirculation parameters and EF measured on the wrist for three groups (see Table 2): Control, CHD and AF. The mean values and standard deviations of the data are presented.

Parameter	Control (N = 17)	CHD ^1^ (N = 11)	AF ^2^ (N = 19)
Capillary blood velocity (µm/s)	1861 ± 1144	1143 ± 603	880 ± 601
Blood aggregate size (µm^2^)	0 ± 0	104 ± 30	100 ± 22
Number of aggregates per running mm	0 ± 0	2618 ± 1904	3259 ± 864
Number of blood aggregates per min	0 ± 0	42 ± 37	68 ± 64
EF ^3^ measured on the wrist (%)	38 ± 26	3 ± 30	19 ± 36

^1^ coronary heart disease; ^2^ atrial fibrillation; ^3^ endothelial function.

## Data Availability

The raw data with all of the identifying information about patients or volunteers removed is available at the following link: https://disk.yandex.ru/i/W8wouI-H8BZlag (accessed on 1 October 2023).

## Supplementary Materials

The following supporting information can be downloaded at: https://www.mdpi.com/article/10.3390/life13102043/s1, Video S1: The capillaries of patient with CHD; Video S2: The capillaries of healthy individual; Figure S1: The blood aggregate size for three groups: Control (healthy individuals), CHD (coronary heart disease) patients and AF (atrial fibrillation) patients; Figure S2: The scatterplot and correlation between the capillary blood velocity and the age of donors in the Control group; Figure S3: The scatterplot and correlation between the capillary blood velocity and the blood aggregate size for two groups of patients: CHD (coronary heart disease) and AF (atrial fibrillation); Figure S4: The scatterplot and correlation between the blood aggregate size and the number of aggregates per running mm for two groups of patients: CHD (coronary heart disease) and AF (atrial fibrillation); Figure S5: The scatterplot and correlation between the blood aggregate size and the number of blood aggregates per min for two groups of patients: CHD (coronary heart disease) and AF (atrial fibrillation); Figure S6: The scatterplot and correlation between the capillary blood velocity and the EF (endothelial function) measured on the wrist for two groups of patients: CHD (coronary heart disease) and AF (atrial fibrillation); Figure S7: The scatterplot and correlation between the blood aggregate size and the EF (endothelial function) measured on the wrist for two groups of patients: CHD (coronary heart disease) and AF (atrial fibrillation).
