# Supplementary material for: Correlation between the Capillary Blood Flow Characteristics and Endothelium Function in Healthy Volunteers and Patients Suffering from Coronary Heart Disease and Atrial Fibrillation: A Pilot Study
Source: Life (Basel). 2023 Oct 12;13(10):2043. doi: 10.3390/life13102043 (PMC10608205; doi:10.3390/life13102043)
Supplement: Supplementary file 1 [file life-13-02043-s001.zip › Supplementary Materials.pdf]

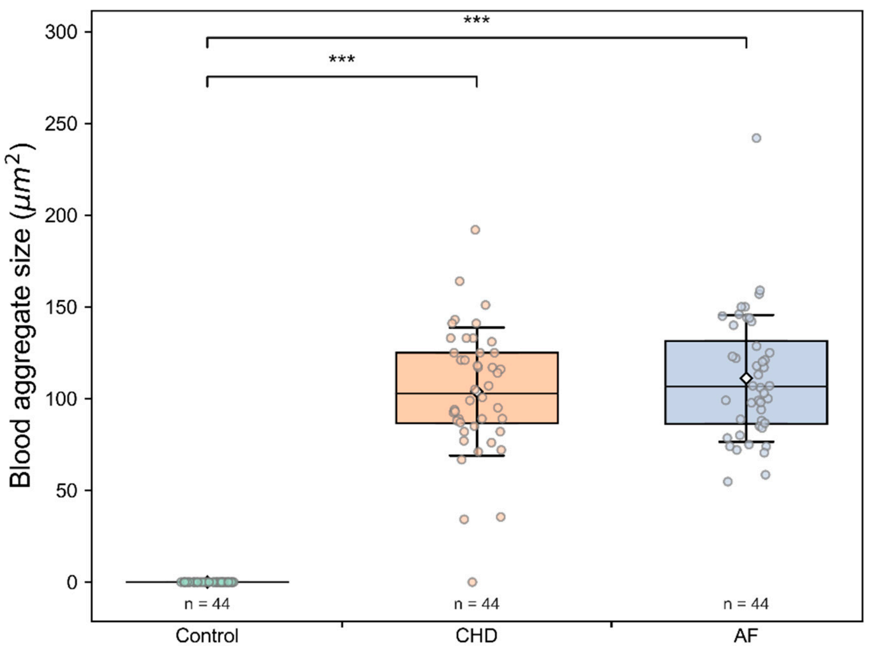

Figure S1. The blood aggregate size for 3 groups: Control (healthy individuals), CHD (coronary heart disease) patients and AF (atrial fibrillation) patients. Each point in the figure corresponds to the average value for a single donor or patient. The box corresponds to the first (Q1) and to the third quartile (Q3) of the data with a median line. The whiskers are standard deviation. The rhombus point is the mean value. **p<0.01; ***p<0.001.

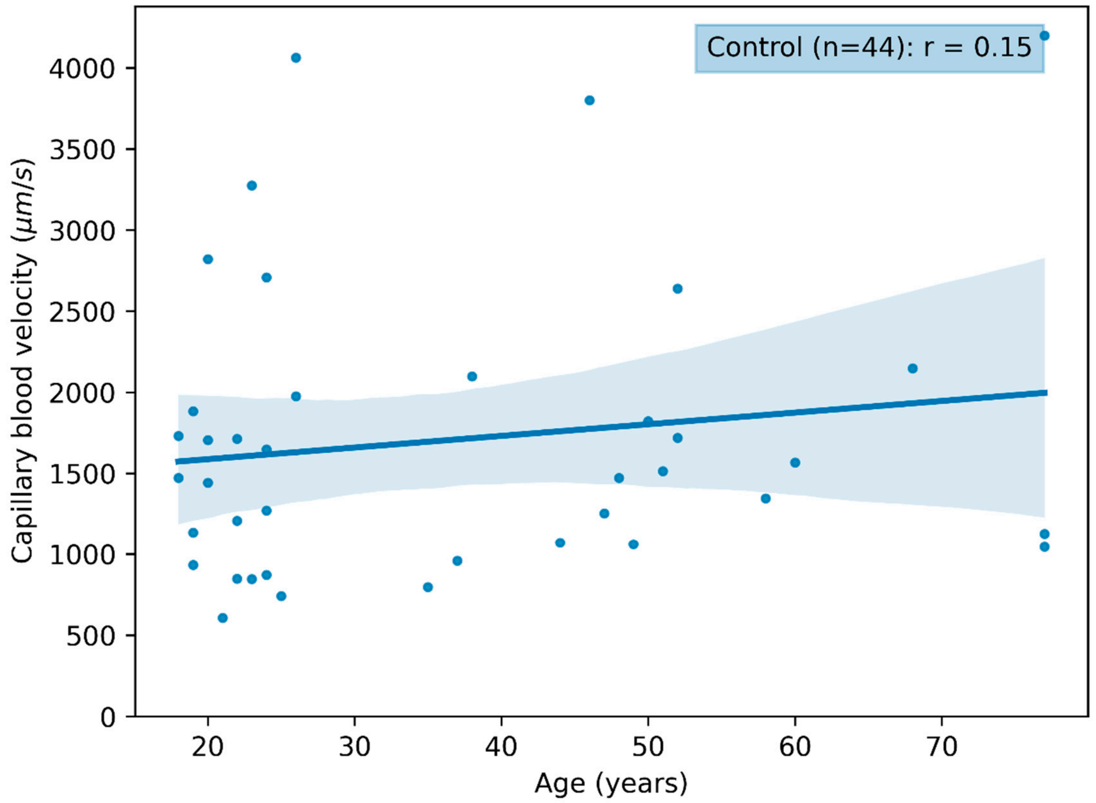

Figure S2. The scatterplot and correlation between the capillary blood velocity and the age of donors in the Control group. Each point in the figure corresponds to the average value for a single donor or patient. The linear regression of the data and its errors for each group are presented. The corresponding Pearson's correlation coefficients are calculated and given in the legend.

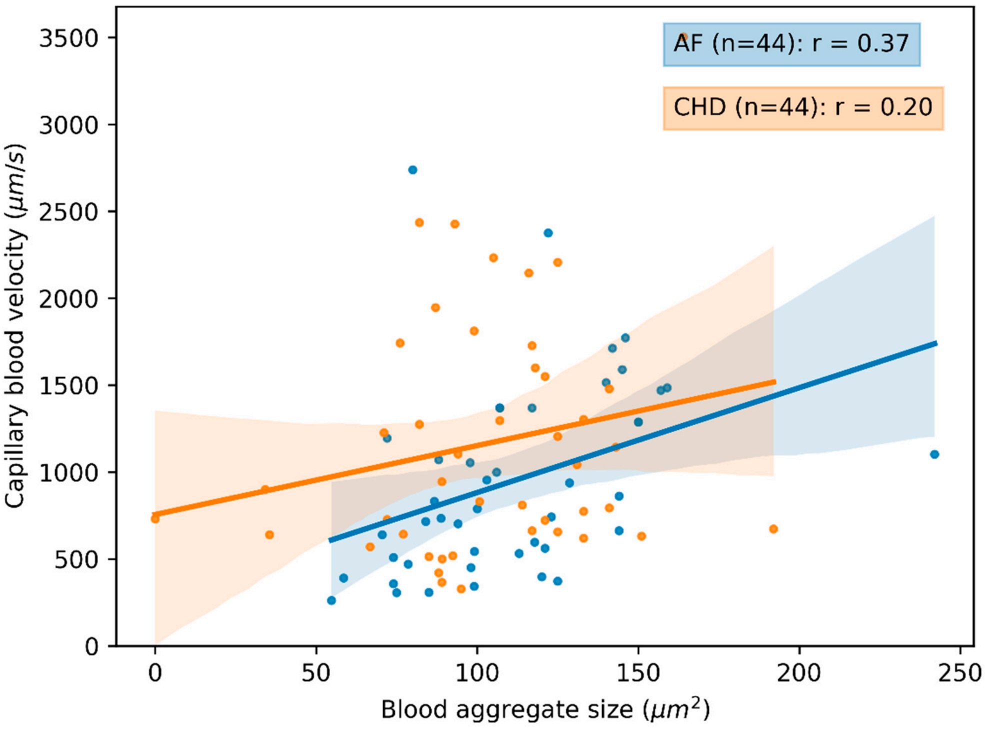

Figure S3. The scatterplot and correlation between the capillary blood velocity and the blood aggregate size for 2 groups of patients: CHD (coronary heart disease) and AF (atrial fibrillation). Data for volunteers in the Control group are omitted due to the absence of observed aggregates in the bloodstream. Each point in the figure corresponds to the average value for a single donor or patient. The linear regression of the data and its errors for each group is presented. Pearson's correlation coefficient is calculated for each group and given in the legend.

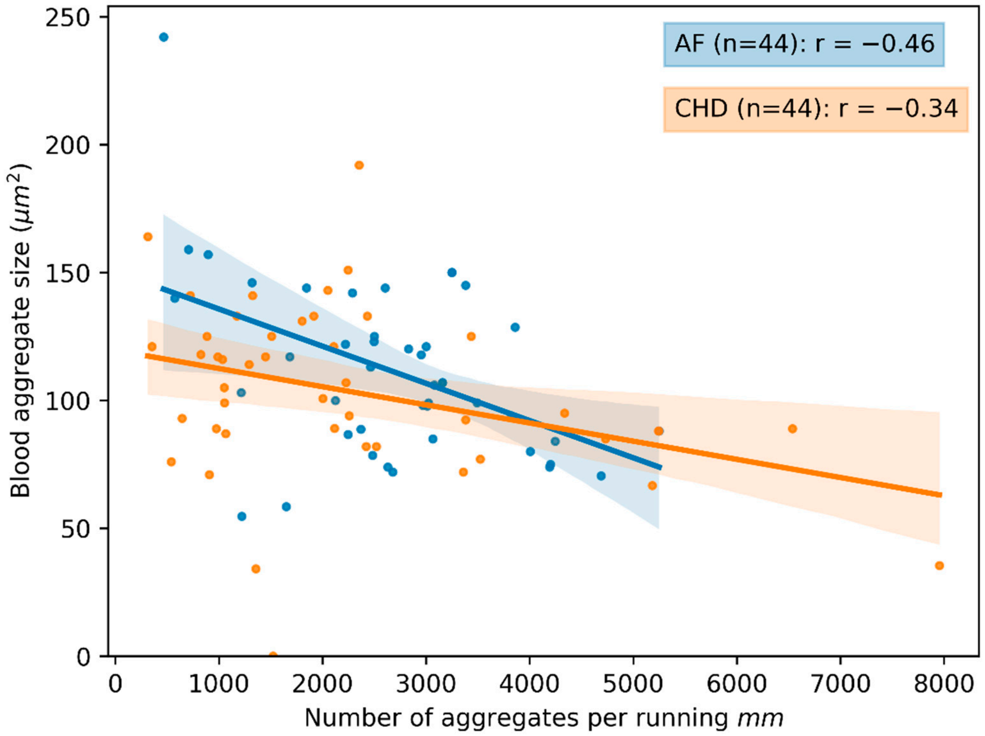

Figure S4. The scatterplot and correlation between the blood aggregate size and the number of aggregates per running mm for 2 groups of patients: CHD (coronary heart disease) and AF (atrial fibrillation). Data for volunteers in the Control group are omitted due to the absence of observed aggregates in the bloodstream. Each point in the figure

corresponds to the average value for a single donor or patient. The linear regression of the data and its errors for each group is presented. Pearson's correlation coefficient is calculated for each group and given in the legend.

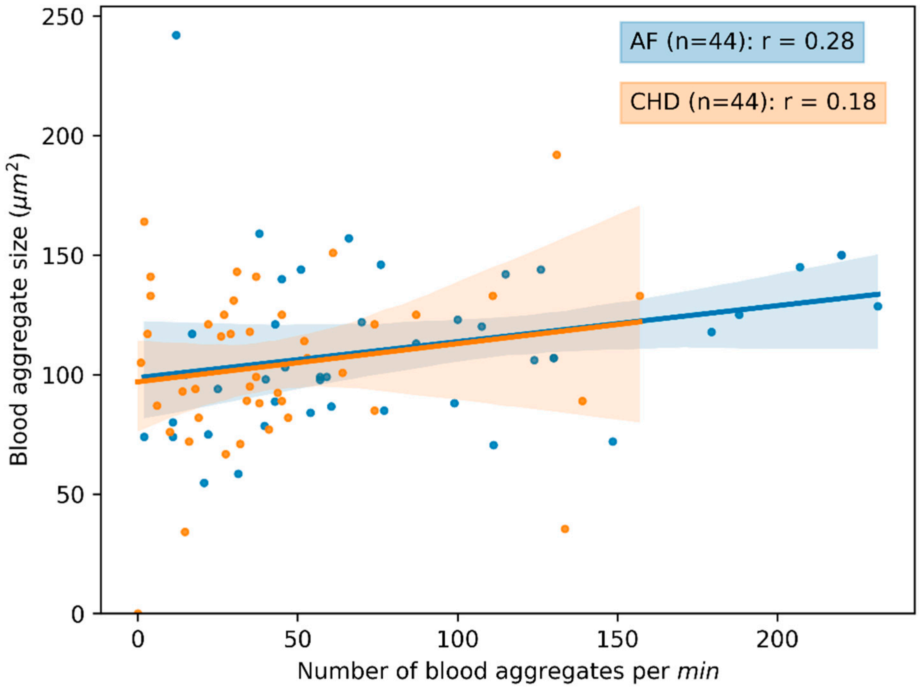

Figure S5. The scatterplot and correlation between the blood aggregate size and the number of blood aggregates per min for 2 groups of patients: CHD (coronary heart disease) and AF (atrial fibrillation). Data for volunteers in the Control group are omitted due to the absence of observed aggregates in the bloodstream. Each point in the figure corresponds to the average value for a single donor or patient. The linear regression of the data and its errors for each group is presented. Pearson's correlation coefficient is calculated for each group and given in the legend.

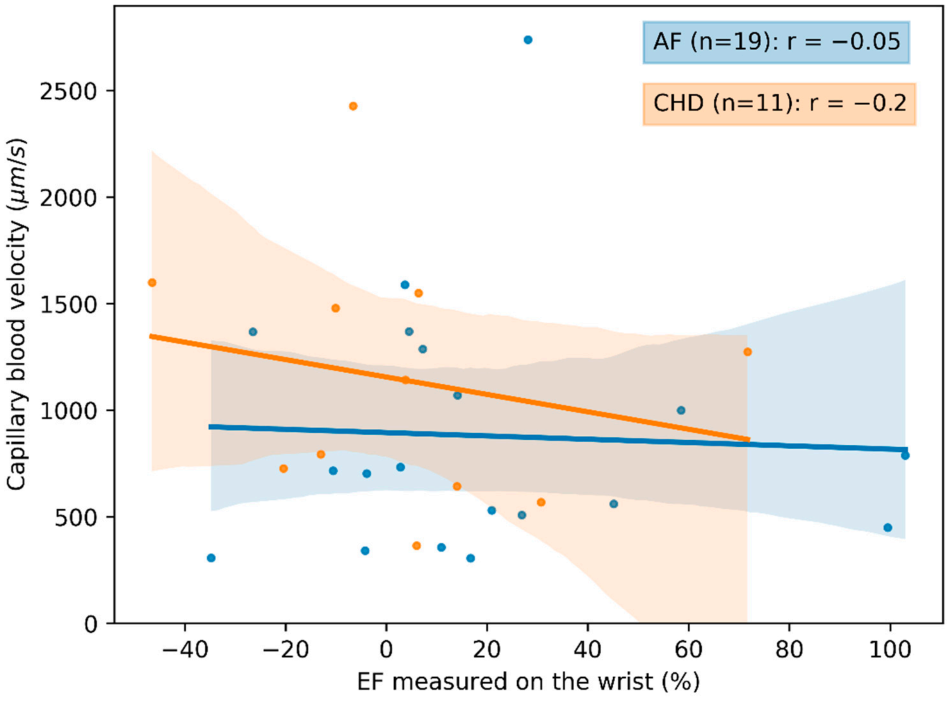

Figure S6. The scatterplot and correlation between the capillary blood velocity and the EF (endothelial function) measured on the wrist for 2 groups of patients: CHD (coronary heart disease) and AF (atrial fibrillation). Data for volunteers in the Control group are omitted due to the absence of observed aggregates in the bloodstream. Each point in the figure corresponds to the average value for a single donor or patient. The linear regression of the data and its errors for each group is presented. Pearson's correlation coefficient is calculated and given in the legend.

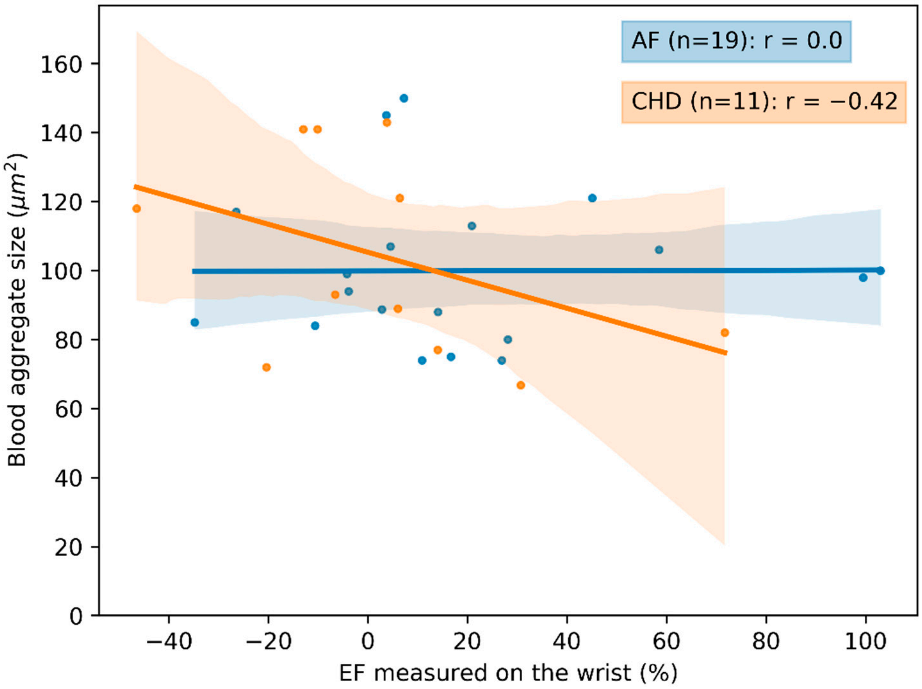

Figure S7. The scatterplot and correlation between the blood aggregate size and the EF (endothelial function) measured on the wrist for 2 groups of patients: CHD (coronary heart disease) and AF (atrial fibrillation). Data for volunteers in the Control group are omitted due to the absence of observed aggregates in the bloodstream. Each point in the figure corresponds to the average value for a single donor or patient. The linear regression of the data and its errors for each group is presented. Pearson's correlation coefficient is calculated and given in the legend.
